# Resistance of *Pseudomonas aeruginosa* to Antibiotics During Long-Term Persistence in Patients with Cystic Fibrosis

**DOI:** 10.3390/antibiotics14030302

**Published:** 2025-03-14

**Authors:** Natalia Belkova, Uliana Nemchenko, Elizaveta Klimenko, Nadezhda Smurova, Raisa Zugeeva, Marina Sukhoreva, Viacheslav Sinkov, Evgenij Savilov

**Affiliations:** 1 Federal State Budgetary Scientific Institution ‘Scientific Centre for Family Health and Human Reproduction Problems’, Epidemiology and Microbiology Institute, 3, K. Marks Str., 664003 Irkutsk, Russia; umnemch@mail.ru (U.N.); klimenko.elizabet@gmail.com (E.K.); nadinasmurova@mail.ru (N.S.); raya.zugeeva@mail.ru (R.Z.); vsinkov@yandex.ru (V.S.); savilov47@gmail.com (E.S.); 2 Regional State Autonomous Healthcare Institution ‘Ivano-Matreninskaya City Children’s Clinical Hospital’, 57, Sovetskaya Str., 664009 Irkutsk, Russia; imdkb-bak@mail.ru

**Keywords:** *Pseudomonas aeruginosa*, cystic fibrosis, antibiotic resistance, whole-genome sequencing, multilocus sequence typing

## Abstract

*Pseudomonas aeruginosa* is one of the leading causes of nosocomial respiratory tract infections, significantly affecting morbidity and mortality. It can persist in the lungs of patients with cystic fibrosis (CF) for extended periods because of its adaptive capacity. The main aim of this study was to determine the phenotypic and genotypic resistance to antibiotics of clinical isolates of *P. aeruginosa* that persist in patients with CF receiving long-term antimicrobial therapy. The study included nine strains of *P. aeruginosa* isolated from the sputum of patients with CF admitted to the hospital. Susceptibility to antibiotics was determined using the European Committee on Antimicrobial Susceptibility Testing (EUCAST) criteria. Whole-genome sequencing was performed for phylogeny, sequence typing, and to identify antibiotic-resistant genes. The study showed that during long-term persistence in the lungs of patients receiving antibacterial therapy, the restoration of susceptibility to antibiotics occurred in some cases. Multilocus sequence typing and phylogeny revealed six sequence types. Functional annotation identified 72 genes responsible for resistance to antibacterial and chemical substances, with either chromosomal or plasmid localisation.

## 1. Introduction

The high morbidity associated with persistent multidrug-resistant bacterial infections is a global public health burden. Persistent infections limit therapeutic options, creating a clinical scenario in which antibiotics are ineffective in treating certain infections. This represents a significant mechanism contributing to widespread antibiotic resistance in infectious agents [1,2,3]. Considering antibiotics as one of the environmental stress factors to which infectious agents are exposed, resistance and persistence can be defined as two physiological systems that enable them to adapt to changing conditions [1]. A resistant cell exhibits genetically mediated survival in a stressful environment, whereas a persistent cell is genetically susceptible to antibiotics but survives by slowing its metabolism, resuming growth once the stress is relieved [3]. These persistent cells are largely responsible for the challenges in treating chronic and recurrent infections. Biofilms, which harbour the highest number of persistent cells, provide protective and barrier functions, enhancing tolerance to stress factors [2].

Cystic fibrosis (CF) is a hereditary disease affecting the glands that secrete external substances, manifesting in pathological conditions of the gastrointestinal tract and respiratory system. The increased viscosity of glandular secretions leads to a chronic inflammatory process in the lungs, exocrine pancreatic insufficiency, hepatobiliary pathology, and an abnormally high electrolyte content in sweat. The respiratory disease associated with CF is characterised by chronic infection and inflammation driven by neutrophils, resulting in progressive airway damage and early mortality.

*Pseudomonas aeruginosa* is one of the leading causes of nosocomial respiratory tract infections, significantly impacting morbidity and mortality and necessitating early detection and aggressive antibiotic treatment [4,5,6]. Experimental data indicate that *P. aeruginosa* is detected in the lower respiratory tract of children with CF under the age of 4 years with a frequency of 31%, while among patients over 18 years of age, the prevalence ranges from 60% to 80% of cases [7]. According to the Cystic Fibrosis Foundation Patient Registry, infection rates with multidrug-resistant *P. aeruginosa* are highest among older adolescents and adults with CF, reflecting the cumulative effects of antibiotic exposure [8].

*Pseudomonas aeruginosa* can persist in the lungs of patients with CF for extended periods because of its strong adaptive capacity [9]. In this regard, the stability and constancy of the microorganism population, as well as changes in the properties of the pathogen that may necessitate adjustments in treatment strategies, are of particular interest [10]. It has been shown that the variability of infectious agents in CF occurs through three mechanisms: phenotypic heterogeneity; microevolution, resulting from the acquisition of mobile genetic elements by the pathogen genome and variability in chromosomal genes; and changes in the genotype of the pathogen within the same species, or the emergence of a new strain or species [10]. Based on findings from various longitudinal studies, the key genetic adaptations of *P. aeruginosa* to the CF lung environment have been summarised, and the phenotypes of these CF-adapted *P. aeruginosa* that contribute to persistent infection have been described [9].

The primary aim of the present study was to determine the genetic variability of clinical isolates of *P. aeruginosa* that persist in patients with CF undergoing continuous antimicrobial therapy.

## 2. Results

### 2.1. Evaluation of the Susceptibility of Pseudomonas aeruginosa Strains to Antibiotics: Phenotypic Aspect

This study included strains of *P. aeruginosa* isolated from paediatric patients aged 8 to 12 years who were hospitalised for treatment of CF. All strains obtained from patients via sputum culture were analysed. All patients received a course of antibiotic therapy according to the recommended regimens both in hospital and after discharge.

Nine strains of *P. aeruginosa* were obtained (Table 1). In one patient (P1), strains were collected at the initiation of treatment and again after 5 months. From another patient (P2), four strains were obtained—at the start of treatment and after 2, 7, and 11 months while receiving antibiotic therapy. Three additional strains were obtained from different patients (P3, P4, and P5) during antibiotic therapy.

Strains isolated at different times from patients with CF exhibited variations in phenotypic properties. In the pair of strains obtained from P1, strain IMB25 was resistant to carbapenems (meropenem) and aminoglycosides (amikacin), but showed susceptibility with increased exposure to cephalosporins (ceftazidime), penicillins (piperacillin/tazobactam), and fluoroquinolones (ciprofloxacin). By contrast, strain IMB100, isolated 5 months later, was susceptible to almost all antibiotics except piperacillin/tazobactam.

Strains isolated from P2 exhibited a similar spectrum of antibiotic susceptibility and were either susceptible or not susceptible with increased exposure to all antibiotics used, except for meropenem. The first strain, IMB54, was resistant to this antibiotic; however, the subsequent isolates—IMB82, IMB102, and IMB105—later obtained from this patient, were all susceptible to meropenem.

Strains isolated from the three other patients (P3, P4, and P5) while undergoing antibiotic therapy were also either susceptible or not susceptible with increased exposure to all antibiotics, except for piperacillin/tazobactam. Strain IMB101 was resistant to piperacillin/tazobactam, whereas strains IMB103 and IMB104 were susceptible with increased exposure.

Thus, it is worth noting that during long-term persistence in patients undergoing antibacterial therapy, a restoration of antibiotic susceptibility was observed in some cases. However, the analysis of phenotypic manifestations alone does not provide sufficient insight into the duration of *P. aeruginosa* persistence in patients with CF or the variability of its biological properties.

### 2.2. Whole-Genome Sequencing of Pseudomonas aeruginosa Strains: Analysis of Draft Genomes and Genotyping

The sequencing of *P. aeruginosa* genomes yielded between 13,477,896 and 50,189,120 reads, with a mean whole-genome coverage of 1328 ± 896-fold. The draft genomes consisted of 249.5 ± 13.72 contigs and contained between 5493 and 5935 coding DNA sequences (CDSs), with a mean of 5522.5 ± 25.48 (Table 2). The GC content in the genomes of all strains was 66%, except for strain IMB104, in which it was 65%. The number of rRNA genes was 12 in most strains, except for IMB101 and IMB104, where 11 rRNA genes were identified. The number of tRNA genes varied significantly, ranging from 64 to 72.

Based on multilocus sequence typing (MLST) (Table 2), six different sequence types were identified. Strains IMB25 and IMB100 (from P1) were assigned to sequence type ST1641, while strains IMB101 (P3), IMB103 (P4), and IMB104 (P5) were classified as ST554, ST379, and ST970, respectively. Strains obtained from P2 were assigned to different sequence types, deviating from the typical pattern of ‘one patient—*P. aeruginosa* strains of a single sequence type’. During the first isolation, strain IMB54 was classified as ST532; however, in subsequent isolations, all three strains—IMB82, IMB102, and IMB105—were assigned to ST555.

**Figure 1:**
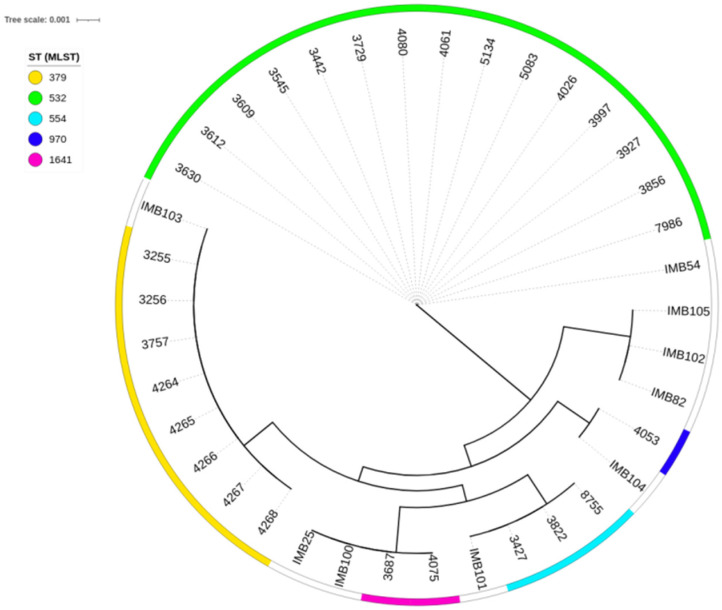
Minimum-spanning trees from allelic profiles obtained for the studied *Pseudomonas aeruginosa* strains and reference genomes of the corresponding sequence types. The labelling of the reference genomes in the tree corresponds to the IDs of these genomes in the PubMLST database.

Phylogenetic analysis revealed that the studied genomes clustered with the reference genomes of the corresponding sequence types (Figure 1). Notably, at the time of analysis, there were no validated genomes of *P. aeruginosa* ST555 in the PubMLST database; therefore, in the phylogenetic tree, this sequence type is represented solely by the genomes obtained in the present study.

Significant differences in the plasmid profiles of the strains were identified (Table 2). The number of plasmids per strain varied from zero to three. The most striking case was observed in P2, with strains classified as ST555. In the genome of strain IMB82, obtained during the second isolation, two plasmids (AA531 and AE982) were identified; however, in subsequent isolations, plasmids were not detected in the genomes of IMB102 and IMB105. Both strains isolated from P1 carried three plasmids in their genomes, while strains from P3, P4, and P5 contained two, zero, and one plasmid, respectively. It is worth noting that with long-term persistence under antibacterial therapy, a reduction in the genome size of clinical strains of *P. aeruginosa* was observed, likely due to the elimination of plasmids.

### 2.3. Evaluation of Susceptibility of Pseudomonas aeruginosa Strains to Antibiotics: Functional Annotation of Genomes

Based on the results of functional annotation, 72 genes responsible for resistance to antimicrobial and chemical substances were identified. The following AMR gene families were detected in the genomes of all analysed strains (see Supplementary Table S1 for details):

- Aminoglycoside-modifying enzymes: APH(3′) (*aph(3*′*)-IIb*);
- Chloramphenicol resistance: chloramphenicol acetyltransferase (*catB7*);
- Fosfomycin resistance: fosfomycin thiol transferase (*fosA*);
- Beta-lactam resistance: *blaPAO*;
- Major facilitator superfamily antibiotic efflux pump: *bcr-1*;
- Small multidrug resistance antibiotic efflux pump: *emrE*;
- Pmr phosphoethanolamine transferase: *arnA*, *arnT*, *basS*, *cprR*, and *cprS*;
- Resistance–nodulation–cell division (RND) antibiotic efflux pump: *cpxR*, *mexA-BCDEFGHIJKLNPQRSTVWY*, *muxABC*, *nalCD*, *opmBDEH*, *oprJM*, *rsmA*, *triABC*, *Type A nfxB*, and *yajC*;
- Outer membrane porin: *parRS*;
- PDC beta-lactamase: *PDC-3*, *PDC-1*, and *PDC-59*;
- Multidrug and toxic compound extrusion transporter: *pmpM*;
- ATP-binding cassette antibiotic efflux pump;
- Major facilitator superfamily antibiotic efflux pump;
- RND antibiotic efflux pump: *soxR*;
- Glycopeptide resistance gene cluster: *vanW* gene in the *vanG* cluster.

Antibiotic resistance is achieved through various mechanisms targeting different classes of antibiotics and chemical compounds. For example, the inactivation of aminoglycoside antibiotics occurs through the involvement of the *aph(3*′*)-IIb* gene, the inactivation of phenicol antibiotics with the *catB7* gene, inactivation of phosphonic acid antibiotics with the *fosA* gene, and inactivation of monobactam, carbapenem, and cephalosporin antibiotics with the *PDC-3*, *PDC-1*, or *PDC-59* genes. In the genomes of clinical strains, the *arnAT* and *vanW* genes (in the *vanG* cluster) were identified, both of which contribute to target alteration—*arnAT* for peptide antibiotics and *vanW* for glycopeptide antibiotics.

Genes such as *basS*, *cprRS*, *mexR*, and *soxR* contribute not only to antibiotic target alteration, but also to antibiotic efflux. *parR* and *parS* are involved in both reduced antibiotic permeability and antibiotic efflux. Notably, the largest number of genes play a role in the efflux mechanism, which affects not only antibiotics, but also other chemicals, such as disinfectants and antiseptics. These genes include *mexGHIJKLMPQVWYZ*, *opmDEH*, *oprMN*, *parRS*, *pmpM*, *soxR*, *triABC*, and *yajC*.

The differences in genotypic antibiotic resistance among strains are summarised in Table 3. The main distinction was the identification of the *blaOXA* gene loci, with strains of different sequence types exhibiting distinct allelic variants of this locus. Sequence type (ST)-dependent allelic variants of the *PDC* and *armR* genes were also identified. All genes were chromosomally located. Additionally, the *adeF* gene was found in the chromosome of the IMB103 strain carrying ST379, whereas *crpP* was localised in the chromosomes of IMB54, IMB101, and IMB104, which carry ST532, ST554, and ST970, respectively.

We note that the *armA* gene was identified in the plasmid of the IMB25 strain, which was obtained during the first isolation from P1. However, in the genome of the IMB100 strain, isolated from the same patient 5 months later, this gene was not detected—neither through search services using online databases nor via BLAST v2.16.0 analysis. A similarly thorough search was conducted to identify the *kpnF* gene, which encodes the *KpnF* subunit of the *KpnEF* protein in the *Klebsiella pneumoniae* genome. In the genome of the *P. aeruginosa* IMB100 strain, this gene was found to have chromosomal localisation. It appears that during persistence within the polymicrobial community formed in patients with CF undergoing long-term antibiotic therapy, horizontal gene transfer and genetic exchange occur, facilitating the development of stable resistance to AMP.

The genes *aph(6)-Id*, *aph(3*″*)-Ib*, *aac(6*′*)-Ib9*, *dfrB4*, *fosA8*, and *sul1* were identified in the IMB25 genome and had plasmid localisation. Notably, the genes *qacEdelta1* and *qacJ*, which confer resistance to antiseptic and disinfectant agents such as quaternary ammonium compounds (e.g., benzalkonium chloride and chlorhexidine) were also identified. These genes had plasmid localisation and were detected only in the strains isolated during the first and second sampling from P1 and P2—IMB25 and IMB82, respectively. The *qacEdelta1* and *qacJ* genes were localised in the AE788 and AE982 plasmids within the genomes of strains IMB25 and IMB82, respectively.

## 3. Discussion

In this study, we compared strains isolated at different times from five patients with CF who were receiving continuous antibacterial treatment. Bacterial cells employ various mechanisms to survive in adverse environmental conditions. With prolonged exposure to antibiotics, cells can not only develop resistance, but also utilise another mechanism: persistence. Unlike a resistant cell, a persistent cell is genetically susceptible to antibiotics but is phenotypically tolerant, exhibiting reduced metabolism and becoming metabolically inactive [1,2,3]. This phenotypic heterogeneity in chronically colonising *P. aeruginosa* populations is well documented and complicates direct comparisons of phenotypic properties between specific strains [4,11]. The phenomenon of persistence has been observed in both Gram-positive and Gram-negative bacteria under the influence of various classes of antibiotics [1]. Studies have shown that the frequency of persistent cell formation depends on the bacterial growth phase and is higher in the stationary phase, during which cells experience increased stress due to high population density, the reduced availability of nutrients, and the presence of additional stress factors such as active secondary metabolites [1]. The passive aspect of persistence should also be noted, as a bacterial cell in response to an antibiotic may exhibit no growth, remain unresponsive, and enter a dormant state without modifying or resisting the antibiotic. However, in the absence of an antibiotic, the persistent cell resumes growth and displays a phenotype susceptible to antibiotics. Experimental evidence has demonstrated that a persistent cell can reversibly shift between persistence and active metabolism depending on the environmental conditions, a phenomenon known as ‘resuscitation’ [12].

We demonstrated that strains isolated from a single patient exhibit different phenotypic antibiotic susceptibility properties. For example, strains IMB25 and IMB100, isolated from the same patient 5 months apart, differed in their resistance to penicillins (piperacillin/tazobactam), carbapenems (meropenem), and aminoglycosides (amikacin). Moreover, upon repeated isolation from the same patient, both the emergence of resistance to antibiotics such as piperacillin/tazobactam and the restoration of susceptibility to antibiotics such as meropenem and amikacin were observed. Additionally, the recovery of susceptibility to meropenem was noted for strains IMB54 and IMB82, though this may have been due to the different sequence types assigned to these strains. Clinical studies have shown that, in most cases, a single strain of *P. aeruginosa* is isolated from patients with chronic infection and persists over time [13,14,15]. However, even in individuals with CF who are chronically infected with only one strain of *P. aeruginosa*, significant genetic diversity among related colonising strains has been identified [9]. At the genetic level, these microorganisms undergo point mutations, insertions, and even large-scale deletions, leading to the development of distinct clonal lineages that compete within the complex environment of the lower respiratory tract in patients with CF [16]. As a result, clonal isolates of the same strain from individual patients exhibit phenotypic heterogeneity [17].

We found that strains isolated from a single patient may belong to either the same or different sequence types. The isolation of different sequence types of *P. aeruginosa* from one patient supports a previously proposed adaptation mechanism, in which complete eradication of the pathogen during antibiotic therapy may lead to its replacement by either another sequence type of the same pathogen or a different species altogether [9,10]. Shaginyan et al. analysed 1800 throat swabs and sputum samples from 300 children with CF over a 10-year period (2008–2017) and identified three main patterns of pathogen variability: population heterogeneity, pathogen microevolution, and replacement with another genotype of the same species [10]. The authors highlighted the epidemiological significance of molecular mechanisms driving pathogen variation, primarily due to the ability of strains to form epidemiologically significant clones [10]. Camus et al. reviewed various longitudinal studies that attempted to compare strains from early infections with those adapted to chronic infections [9]. Their findings provide an updated description of the key genetic adaptations of *P. aeruginosa* to the CF lung environment.

Our studies have shown that strains of *P. aeruginosa* isolated from patients with CF undergoing long-term antibiotic therapy, despite exhibiting different phenotypic properties, share similarities in genome structure and the presence of key genes responsible for resistance to antibacterial drugs and chemicals.

Of greatest interest, in our opinion, are the differences observed among the analysed strains in genes with chromosomal localisation, such as *adeF*, *crpP*, *armR*, and *kpnF*. It has been shown that the *adeF* gene is involved in resistance to fluoroquinolone and tetracycline antibiotics through the antibiotic efflux mechanism [18]. AdeF is a membrane fusion protein within the multidrug efflux complex AdeFGH. Overexpression of intrinsic efflux systems, such as RND efflux pumps, is a known mechanism contributing to multidrug resistance in opportunistic bacteria [19,20,21,22,23]. The RND efflux pumps AdeABC and AdeIJK were initially studied in *Acinetobacter baumannii* strains, which are nosocomial pathogens with multidrug resistance [19]. However, the application of next-generation sequencing methods has enabled the identification of homologous regions with both chromosomal and plasmid localisation in the complete genomes of various opportunistic microorganisms, including *P. aeruginosa*, as well as in whole-genome shotgun assemblies [18].

The *crpP* gene was found at a chromosomal localisation in the genomes of three *P. aeruginosa* types: strains with different sequence IMB54 (ST532), IMB101 (ST554), and IMB104 (ST970). This gene is involved in peptide antibiotic resistance through the mechanisms of antibiotic target alteration and antibiotic efflux [24]. There are at least three main mechanisms of quinolone resistance in bacteria: mutations in genes encoding quinolone-targeted proteins, changes in the expression of efflux pumps or porin channels, and the acquisition of quinolone resistance genes such as *crpP* [25]. The CrpP enzyme, capable of phosphorylating ciprofloxacin (CIP), was first described in 2018 [26]. It has been experimentally demonstrated that the pUM505 plasmid, isolated from a clinical strain of *P. aeruginosa* carrying the *crpP* gene, confers resistance to CIP when transferred to the *P. aeruginosa* PU21 strain. When the *P. aeruginosa* PAO1 strain was transformed with this plasmid, the transformant exhibited a fourfold increase in MIC values for CIP, norfloxacin, and moxifloxacin compared with the receptor strain *P. aeruginosa* PAO1 [26]. Whole-genome sequencing has revealed a high diversity of *crpP* homologues in the genomes of *P. aeruginosa* [27,28,29,30,31,32,33]. Notably, clinical strains of *P. aeruginosa* carrying the *crpP* gene have been isolated from various biotopes, including different parts of the respiratory tract [32,34,35]. Currently, homologous variants are being actively described, and data are being systematised for the typing and nomenclature of *crpP* variants in genomes, as well as in integrative and conjugative elements carrying *crpP* [31,33]. The typing of *crpP* variants, their geographic distribution, presence in mobile genetic elements, and potential ancestral origin are of great interest in epidemiological studies of antibiotic resistance.

The *armR* gene is part of the system regulating the formation of the MexR-ArmR complex and plays a role in controlling the expression of the *mexAB-oprM* operon—one of the four efflux systems of the RND family—contributing to multidrug resistance [23,36]. Experimental studies highlight the key role of genes in the NalC regulatory pathway (*nalC*, *armR*, and *mexR*), as well as *AmgRS*, in providing protection against aldehyde biocides, underscoring their importance in *P. aeruginosa* resistance [37,38]. Notably, the *armR* gene has been proposed as part of a panel of molecular markers for analysing *P. aeruginosa* clonal lineages to assess their virulence and antibiotic resistance [39,40]. The detection of markers associated with identifying high-risk clones during molecular surveillance necessitates additional precautions for infection control.

Equally noteworthy is the chromosomal localisation of the *kpnF* gene in the genome of the IMB100 strain, which was repeatedly isolated from P1, but was not detected via any of the tested methods in the genome of the IMB25 strain obtained during the first isolation. This suggests that horizontal gene transfer occurred within a heterogeneous microbial community during persistent infection in a patient with CF. The *kpnF* gene encodes the KpnF subunit of the KpnEF protein, which is homologous to the EbrAB protein of *Escherichia coli* and performs similar functions [41]. KpnEF belongs to the small multidrug resistance family of efflux pumps [41]. The direct involvement of *kpnEF* in capsule synthesis in mucoid strains of *Klebsiella pneumoniae* has been demonstrated, with mutations in KpnEF leading to increased susceptibility to antibiotics such as cefepime, ceftriaxone, colistin, erythromycin, rifampin, tetracycline, and streptomycin. Additionally, mutations resulted in heightened sensitivity to structurally related chemicals, such as sodium dodecyl sulphate, deoxycholate, dyes, benzalkonium chloride, chlorhexidine, and triclosan, as well as increased vulnerability to hyperosmotic conditions and high concentrations of bile [41].

The main limitation of our study was the small number of strains that we were able to analyse. In the year of the study, we received only nine isolates from patients with CF in a paediatric hospital. We will continue to select strains from patients with CF to identify genetic determinants that distinguish them from other clinically significant isolates of *P. aeruginosa*. Another important aspect of future studies could be the comparison of *P. aeruginosa* genomes that circulate in different hospitals with the same profile, or in the hospital from patients with different diseases.

## 4. Materials and Methods

### 4.1. Strain Collection and Identification

The study examined nine clinical strains of *P. aeruginosa* from the culture collection of the Laboratory of Microbiome and Microecology at the Institute of Epidemiology and Microbiology of the Scientific Centre for Family Health and Human Reproduction Problems [42]. The strains were obtained from patients with CF aged 8 to 12 years who were treated at the Ivano-Matreninskaya City Children’s Clinical Hospital (Irkutsk, Russia).

The strains were isolated from the sputum of hospitalised patients with CF. Identification was performed using a bacteriological method that assessed the morphological, cultural, tinctorial, and biochemical properties of the isolates, with confirmation provided via MALDI-TOF direct protein profiling [43].

### 4.2. Antibiotic Susceptibility Testing

Antibiotic susceptibility was determined using standard methods based on the criteria of the European Committee on Antimicrobial Susceptibility Testing (EUCAST), version 11.0 (valid from 1 January 2021) and version 12.0 (valid from 1 January 2022) [44]. The *P. aeruginosa* type strain ATCC 27853 was used for quality control. The study included discs containing piperacillin–tazobactam (30–6 µg), ceftazidime (10 µg), meropenem (10 µg), amikacin (30 µg), and ciprofloxacin (5 µg) (HiMedia Laboratories Pvt. Limited, Maharashtra, India; Bio-Rad Laboratories, Hercules, CA, USA).

To assess antibiotic susceptibility, a bacterial suspension was prepared according to the standard method, with an optical density of 0.5 on the McFarland scale. Susceptibility was determined using the disc diffusion method on Mueller–Hinton medium (HiMedia Laboratories Pvt. Limited, Maharashtra, India). Strains were classified as resistant (R), susceptible with increased exposure (I), or susceptible (S) based on the defined criteria.

### 4.3. Whole-Genome Sequencing and Bioinformatical Analyses

The biomass of daily cultures was used for DNA extraction, as described previously [45]. Genomic DNA was isolated using the Quick-DNA Fungal/Bacterial Miniprep Kit (Zymo Research, Tustin, CA, USA). The quality and quantity of DNA were assessed using a Nano-500 spectrophotometer and a Qubit 4 fluorometer with a Qubit ds-DNA HS Assay Kit (Invitrogen, Carlsbad, CA, USA). A DNA concentration of >4 ng/μL was considered acceptable for the whole-genome sequencing of the sample.

Whole-genome sequencing of the strains was performed on Illumina NextSeq 550 equipment using the Illumina^®^ DNA Prep Tagmentation, IDT^®^ for Illumina^®^ DNA/RNA UD Indexes Set Tagmentation, and NextSeq 500/550 High Output Kit v2.5 (300 Cycles) library preparation reagent kits, following the manufacturer’s recommendations.

Genome assembly was performed using SPAdes v3.11.1 software [46]. Contig alignment and orientation correction were carried out using MAUVE v2.4.0 [47] and the *P. aeruginosa* PAO1 reference genome (GenBank AE 004091.2).

Strain typing based on genome sequences was performed using the PubMLST database [48], and antibiotic-resistant genes were identified using the ResFinder v4.6.0 database [49], RGI v6.0.3, and CARD v3.3.0 [50,51,52]. The localisation (plasmid or chromosomal) of antibiotic-resistant genes was determined using BLAST+ v2.16.0 [53]. Viral sequences were identified using the PHASTER tool [54].

Functional annotation was performed using Prokka v1.14.6 [55].

To construct the phylogeny, reference strains of *P. aeruginosa* sequence types ST379, ST532, ST554, ST970, and ST1641 from the PubMLST database, along with those obtained during this study, were used. The GrapeTree tool [56] and MLST gene sequences were used to build the tree. The nearest neighbour algorithm was applied to calculate the phylogeny.

## Figures and Tables

**Table 1:** Summary of clinical strains of *Pseudomonas aeruginosa* and results of antibiotic susceptibility assessment. Susceptibility classifications: susceptible (S), indicated in yellow; susceptible with increased exposure (I), indicated in orange; and resistant (R), marked in red.

Indicators	IMB25	IMB100	IMB54	IMB82	IMB102	IMB105	IMB101	IMB103	IMB104
Strain description									
Date of isolation	20 May 2021	15 October 2021	6 August 2021	15 October 2021	29 March 2022	19 July 2022	29 March 2022	29 March 2022	19 July 2022
Patient	P1	P1	P2	P2	P2	P2	P3	P4	P5
Susceptibility to antibiotics									
Piperacillin/tazobactam	I	R	I	I	I	I	R	I	I
Ceftazidime	I	I	I	I	I	I	I	I	I
Meropenem	R	S	R	S	S	S	S	S	S
Ciprofloxacin	I	I	I	I	I	I	I	I	I
Amikacin	R	S	S	S	S	S	S	S	S

**Table 2:** Brief description of the draft genomes of *Pseudomonas aeruginosa* strains: results of assembly, genome annotation, and MLST.

Indicators	IMB25	IMB100	IMB54	IMB82	IMB102	IMB105	IMB101	IMB103	IMB104
Patient	P1		P2				P3	P4	P5
Genome assembly									
Number of reads	50,189,120	36,044,976	37,282,953	33,293,656	5,815,755	13,477,896	12,369,102	3,737,929	14,328,761
Number of scaffolds	235	258	241	264	322	274	121	278	487
N50	67,136	65,489	63,297	56,717	328,046	298,770	255,738	550,311	238,191
Genome annotation									
GC, %	66	66	66	66	66	66	66	66	65
Number of CDSs	5539	5548	5493	5510	5874	5874	5895	5910	5935
Number of rRNA genes	12	12	12	12	12	12	11	12	11
Number of tRNA genes	72	72	70	72	69	69	64	66	64
Number of plasmids	3	3	2	2	0	0	2	0	1
MLST									
ST	1641	1641	532	555	555	555	554	379	970
*acsA*	11	11	5	16	16	16	16	39,345	6
*aroE*	10	10	4	5	5	5	5	5	5
*guaA*	1	1	5	30	30	30	12	11	11
*mutL*	3	3	5	11	11	11	3	28	3
*nuoD*	27	27	5	3	3	3	2	4	4
*ppsA*	4	4	20	20	20	20	15	4	3

**Table 3:** Differences in genotypic antibiotic resistance among clinical strains of *Pseudomonas aeruginosa*. The presence of chromosomal DNA (^ch^) is indicated in yellow, plasmid DNA (^pl^) in orange, and the absence of the gene (nd) in green.

Indicators	IMB25	IMB100	IMB54	IMB82	IMB102	IMB105	IMB101	IMB103	IMB104
Patient	P1		P2				P3	P4	P5
*BlaOXA*	396 *	396	906	486	486	486	494	904	50
*PDC-3*	+^ch^	+^ch^	nd	+^ch^	+^ch^	+^ch^	+^ch^	+^ch^	nd
*PDC-1*	nd	nd	nd	nd	nd	nd	nd	nd	+^ch^
*PDC-59*	nd	nd	+^ch^	nd	nd	nd	nd	nd	nd
*adeF*	nd	nd	nd	nd	nd	nd	nd	+^ch^	nd
*crpP*	nd	nd	+^ch^	nd	nd	nd	+^ch^	nd	+^ch^
*armR*	nd	nd	nd	+^ch^	+^ch^	+^ch^	nd	+^ch^	+^ch^
*kpnF*	nd	+^ch^	nd	nd	nd	nd	nd	nd	nd
*aph(6)-Id*	+^pl^	nd	nd	nd	nd	nd	nd	nd	nd
*aph(3*″*)-Ib*	+^pl^	nd	nd	nd	nd	nd	nd	nd	nd
*aac(6*′*)-Ib9*	+^pl^	nd	nd	nd	nd	nd	nd	nd	nd
*armA*	+^pl^	nd	nd	nd	nd	nd	nd	nd	nd
*dfrB4*	+^pl^	nd	nd	nd	nd	nd	nd	nd	nd
*fosA8*	+^pl^	nd	nd	nd	nd	nd	nd	nd	nd
*sul1*	+^pl^	nd	nd	nd	nd	nd	nd	nd	nd
*qacEdelta1*	+^pl^	nd	nd	nd	nd	nd	nd	nd	nd
*qacJ*	nd	nd	nd	+^pl^	nd	nd	nd	nd	nd

Note: *: beta-lactamase gene name. nd: genes were marked as ‘not detected’ when a search using three databases (ResFinder 4.6.0, RGI 6.0.3, and CARD 3.3.0) showed no results.

## Data Availability

The raw data have been deposited in the NCBI database as Bioproject PRJNA1026796.

## Supplementary Materials

The following supporting information can be downloaded at: https://www.mdpi.com/article/10.3390/antibiotics14030302/s1. Table S1: Genes, responsible for the resistance to antibacterial and chemical substances detected in the genomes of clinic strains of Pseudomonas aeruginosa.
